# Metabolomic Alterations in the Digestive System of the Mantis Shrimp *Oratosquilla oratoria* Following Short-Term Exposure to Cadmium

**DOI:** 10.3389/fphys.2021.706579

**Published:** 2021-08-05

**Authors:** Yingjiang Xu, Huan Liu, Dianfeng Han, Lihua Ren, Xianghong Gong, Fang Jiang, Yanmei Cui, Xiaojing Liu, Chuanbo Ren, Jinglin Xue, Xiuhui Tian

**Affiliations:** ^1^Shandong Key Laboratory of Marine Ecological Restoration, Shandong Marine Resource and Environment Research Institute, Yantai, China; ^2^College of Food Sciences and Technology, Shanghai Ocean University, Shanghai, China

**Keywords:** metabolomics, toxicology, cadmium, lipid metabolism, energy metabolism

## Abstract

Mantis shrimp *Oratosquilla oratoria* is an economically critical aquatic species along the coast of China but strongly accumulates marine pollutant cadmium (Cd) in its digestive system. It is necessary to characterize the toxicity of Cd in the digestive system of mantis shrimp. The metabolic process is an essential target of Cd toxicity response. In this work, we used ultra-performance liquid chromatography coupled with time-of-flight mass spectrometry (UPLC-TOF-MS) for untargeted metabolomics to characterize the metabolic changes in the digestive system of *O. oratoria*, exposed to 0.05 mg/L for 96 h. The aim of this study was to further investigate the effect of *O. oratoria* on Cd response to toxicity and develop biomarkers. Metabolomics analysis showed the alteration of metabolism in the digestive system of mantis shrimp under Cd stress. A total of 91 metabolites were differentially expressed and their main functions were classified into amino acids, phospholipids, and fatty acid esters. The enrichment results of differential metabolite functional pathways showed that biological processes such as amino acid metabolism, transmembrane transport, energy metabolism, and signal transduction are significantly affected. Based on the above results, the Cd-induced oxidative stress and energy metabolism disorders were characterized by the differential expression of amino acids and ADP in mantis shrimp, while the interference of transmembrane transport and signal transduction was due to the differential expression of phospholipids. Overall, this work initially discussed the toxicological response of Cd stress to *O. oratoria* from the metabolic level and provided new insights into the mechanism.

## Introduction

The mantis shrimp *Oratosquilla oratoria* is widely distributed in the coastal areas of the Pacific Northwest (Zhang et al., 2014; Lou et al., 2018). Its high protein content and structurally balanced amino acids (Holmstrand et al., 2006) render it an important economic species along with coastal of China and Southeast Asia (Liu and Cui, 2010; Yan et al., 2018; Li et al., 2020). The development of coastal industries has increased the discharge of pollutants into seawater, and among them trace metals can accumulate in aquatic organisms through the food chain. Compared with trace metals such as copper, chromium, and lead, *O. oratoria* has a more vital ability to accumulate cadmium (Cd; Zhang et al., 2016; Xu et al., 2019; Zhao et al., 2019). The concentrations of trace metals have been shown to be much higher in crustaceans than in fish (Hu et al., 2016; Fu et al., 2017). Wu et al. (2018) conducted the provisional tolerable weekly intake (PTWI) assessment of the content of Cd in mantis shrimp in the northern seas of Fujian and found that there is a higher risk.

Cadmium readily absorbs into and accumulates in the human body and has a low excretion rate, exerting adverse effects on metabolism, growth, and reproduction (Bernard, 2004; Henson and Chedrese, 2004; Godt et al., 2006; Kumar and Sharma, 2019). At the molecular level, Cd causes oxidative stress and DNA damage and induces autophagy (Wang et al., 2008; Gill and Tuteja, 2010; Đukić-Ćosić et al., 2020). Studies of Cd in aquatic organisms typically measure antioxidant enzymes to determine the extent of contamination (Messaoudi et al., 2009). However, Cd toxicity is manifested through multiple interrelated molecular mechanisms that impair metabolic pathways (Đukić-Ćosić et al., 2020), necessitating in-depth analysis combined with species-specific exposure measurements.

Metabolomics has been widely used in the study of plants (Sumner et al., 2003; Mwamba et al., 2020), animals (Ji et al., 2015; Kim et al., 2016; Song et al., 2018), microorganisms (Milreu et al., 2013), and ecosystems (Boroujerdi et al., 2009), as well as in elucidating the toxicity mechanisms of pollutants because it maps alterations in metabolites induced by exposure (Nicholson et al., 1999; Sugiura et al., 2005). The high applicability of metabolomics is because it qualitatively and quantitatively characterizes the chemical characteristics of low-molecular metabolites (<1,000 Da), which are the final products of cell regulatory pathways in cells, tissues (Cappello, 2020; Sun et al., 2020). The expression level of metabolites indicates that it changes with the physiological, developmental, or pathological state of cells, tissues, organs, and even the entire organism (Cappello, 2020). The combination of liquid chromatography (LC) and mass spectrometry (MS) has become a mature technology in metabolomics research due to its sensitivity (Vinaixa et al., 2016; Chaleckis et al., 2019) and has now been applied to the study of metabolic processes of aquatic organisms and the discovery of biomarkers (Venter et al., 2018; Olsvik et al., 2019).

This study investigated the effects of the highly accumulated Cd in *O. oratoria* on the differential expression of different metabolites, and whether it interferes with metabolic pathways such as energy metabolism, oxidative stress, and signal transduction. In the present study, we used ultra-performance liquid chromatography coupled with time-of-flight mass spectrometry (UPLC-TOF-MS) for untargeted metabolomics to record the changes in metabolite levels and perform functional annotations in the digestive system of *O. oratoria* under Cd stress. This research provides a basis for developing strategies for preventing Cd pollution in the habitats of *O. oratoria*.

## Materials and Methods

### Experimental Animals

The seawater Cd concentration reached 5 –16 μg/L in northern China sea (Gao et al., 2014; Ji et al., 2019). In this experiment, the sublethal concentration of 50 μg/L was selected, which is 10 times the seawater standard of China used for aquaculture (Zhang and Zhai, 2019). Choosing the exposure time of 96 h was considered for the method of the acute toxicity. This exposure condition has also been applied in previous studies (Ren et al., 2019; Liu et al., 2021), and the accumulation of pollutants and the expression rate of antioxidant enzymes are significantly different, compared with the control group (CG).

*Oratosquilla oratoria* were purchased from Yangma Island (Yantai, Shandong Province, China) in one batch. After cultivation for 4 days in the laboratory at 18–20°C, 40 ± 10 g *O. oratoria* were selected and randomized into 21 3-L tanks of seawater with four individuals in each tank. The offshore deep underground seawater was gathered as bleeding seawater, with salinity at 30.5 ± 0.8‰ and pH at 7.7 ± 0.3. Nine tanks were used for CGs and 12 tanks were used for the test groups (TGs). Cd (0.75 ml of 2.0 g/L Cd^2+^, Sinopharm Chemical Reagent Co., Ltd., Shanghai, China) was added to 30 L seawater, and the final concentration of Cd^2+^ was controlled to 0.05 mg/L in the test tanks. The exposure duration was 96 h and no feed was provided during the test period. Dead shrimp were removed daily. During the experiment, half of the water was changed every day and the Cd^2+^ solution was supplemented to maintain the experimental concentration. After 96 h, the digestive system tissues (mainly the intestine below the thorax, intestinal glands, and hepatopancreas) were removed and placed in 1.5-ml cryotubes. Each cryotube contained the tissues of two individuals and eight biological replicates were set in each group. The samples are stored at −80°C for testing.

Cadmium concentrations in tissues were determined by inductively coupled plasma mass spectrometry (ICAP-RQ, Thermo Fisher Scientific, Waltham, MA, United States). Wet samples (0.5–1.0 g) were homogenized, mixed with 10 ml analytical-grade nitric acid (Merck, Kenilworth, NJ, United States), and digested in a microwave system (TOPEX, Shanghai Yiyao Instrument Technology Development Company, Shanghai, China). The pressure was set to 20 atm and the samples were heated to 120°C for 5 min, 150°C for 10 min, and 190°C for 20 min.

### Metabolic Sample Preparation

The sample was transferred from −80 to −20°C for metabolite extraction. The following steps were performed by Majorbio Bio-Pharm Technology Co., Ltd. (Shanghai, China) following the method used by Wang et al. (2019). Around 50 mg of solid samples were accurately weighed on ice, and the metabolites were homogenized in 400 μl extraction solution [methanol:water: 4:1 (v/v); −20°C]. The solution was ultrasonically extracted on ice for 10 min and stored at −20°C for 30 min. The mixture was allowed to settle at −20°C. It was treated with a high-throughput tissue crusher (Wonbio-96c, Shanghai Wanbo Biotechnology Co., Ltd.) at 50 Hz for 6 min, followed by vigorous mixing for 30 s and ultrasound treatment at 40 kHz for 30 min at 5°C. After centrifugation at 13,000 *g* at 4°C for 15 min, the supernatants were carefully transferred to sample vials for UPLC-TOF-MS.

Quality control samples were prepared by mixing aliquots of all samples and analyzed as representing the entire sample set. Quality control samples were injected every eight samples to monitor the stability of the analysis process.

### Metabolite Profiling

Chromatographic separation of the metabolites was performed on an AB SCIEX UPLC-TOF-MS system equipped with an ACQUITY BEH C18 column (100 mm × 2.1 mm i.d., 1.7 μm; Waters, Milford, MA, United States). The mobile phases consisted of 0.1% formic acid in water (solvent A) and 0.1% formic acid in an acetonitrile/isopropanol mixture [1:1 (v/v); solvent B]. The solvent gradient was as follows: 0–3 min, 95–80% solvent A; 3–9 min, 20–95% solvent B; 9–13 min, fixed at 95% solvent B; 13–13.1 min, 5–95% solvent A; 13.1–16 min, fixed at 95% solvent A for equilibration. The injection volume was 2 μl and the flow rate was 0.4 ml/min. The column temperature was maintained at 40°C. All samples were stored at 4°C during analysis.

Mass spectrometry data were collected using a time-of-flight mass spectrometer equipped with an electrospray ionization source operating in the positive and negative ion modes. The optimal conditions were as follows: Aus gas heater temperature, 400°C; sheath gas flow rate, 40 psi; Aus gas flow rate, 30 psi; ion-spray voltage floating, −2,800 V in the negative mode (NEG) and 3,500 V in the positive mode (POS); normalized collision energy, 20–40–60 V rolling for tandem mass spectrometry. Data were acquired in the data-dependent acquisition mode and detection was performed over a mass range of 70–1,050 *m/z*.

The raw data were imported into the Progenesis QI 2.3 (Nonlinear Dynamics, Waters, United States) for peak detection and alignment. For preprocessing the raw data, metabolic features detected in at least 80% of any sample set were retained. After filtering, minimum metabolite values were substituted for missing values and each metabolic feature was normalized by sum. The internal standard was used for data reproducibility and metabolic features, for which, the relative SD of the internal standard exceeded 30%. Following normalization and imputation, statistical analysis was performed on log-transformed data to identify significant differences in metabolite levels between comparable groups. The preprocessing results generated a data matrix consisting of the retention time (RT), mass-to-charge ratio (m/z) values, and peak intensity.

Mass spectra of these metabolic features were identified by using the accurate mass, MS/MS fragments spectra and isotope ratio difference with searching in reliable biochemical databases as Human metabolome database (HMDB)^1^ and Metlin database.^2^ For metabolites having MS/MS confirmation, only the ones with MS/MS fragments score above 30 were considered as confidently identified. Otherwise, metabolites had only tentative assignments.

### Multivariate Statistical Analysis

A multivariate statistical analysis was performed on Majorbio Cloud Platform.^3^ An unsupervised principal component analysis (PCA) method was used to obtain an overview of metabolic data and overall clustering, trends, and outliers were visualized. All metabolite variables were adjusted proportionally to unit variance prior to performing PCA. Orthogonal Partial Least Squares Discriminant Analysis (PLS-DA) and Partial Least Squares Discriminant Analysis (OPLS-DA) was used for statistical analysis to determine the overall metabolic changes between comparable groups. All of the metabolite variables were scaled to pareto Scaling before conducting the OPLS-DA. Variable importance in the projection (VIP) were calculated in OPLS-DA and PLS-DA model. *p*-values were estimated with paired Student’s *t*-test on Single dimensional statistical analysis.

### Differential Metabolite Analysis

Statistically significance among groups were selected with VIP value more than 1 and *p*-value less than 0.05. Differential metabolites among two groups were summarized and mapped into their biochemical pathways through metabolic enrichment and pathway analysis based on database search (KEGG).^4^ These metabolites can be classified according to the pathways they were involved or the functions they performed. Enrichment analysis usually analyzes a group of metabolites in a function node, whether it appears or not. The principle was that the annotation analysis of a single metabolite develops into an annotation analysis of a group of metabolites. scipy.stats (Python packages)^5^ was exploited to identify statistically significantly enriched pathways using Fisher’s exact test.

## Results

### Exposure

**Figure 1:**
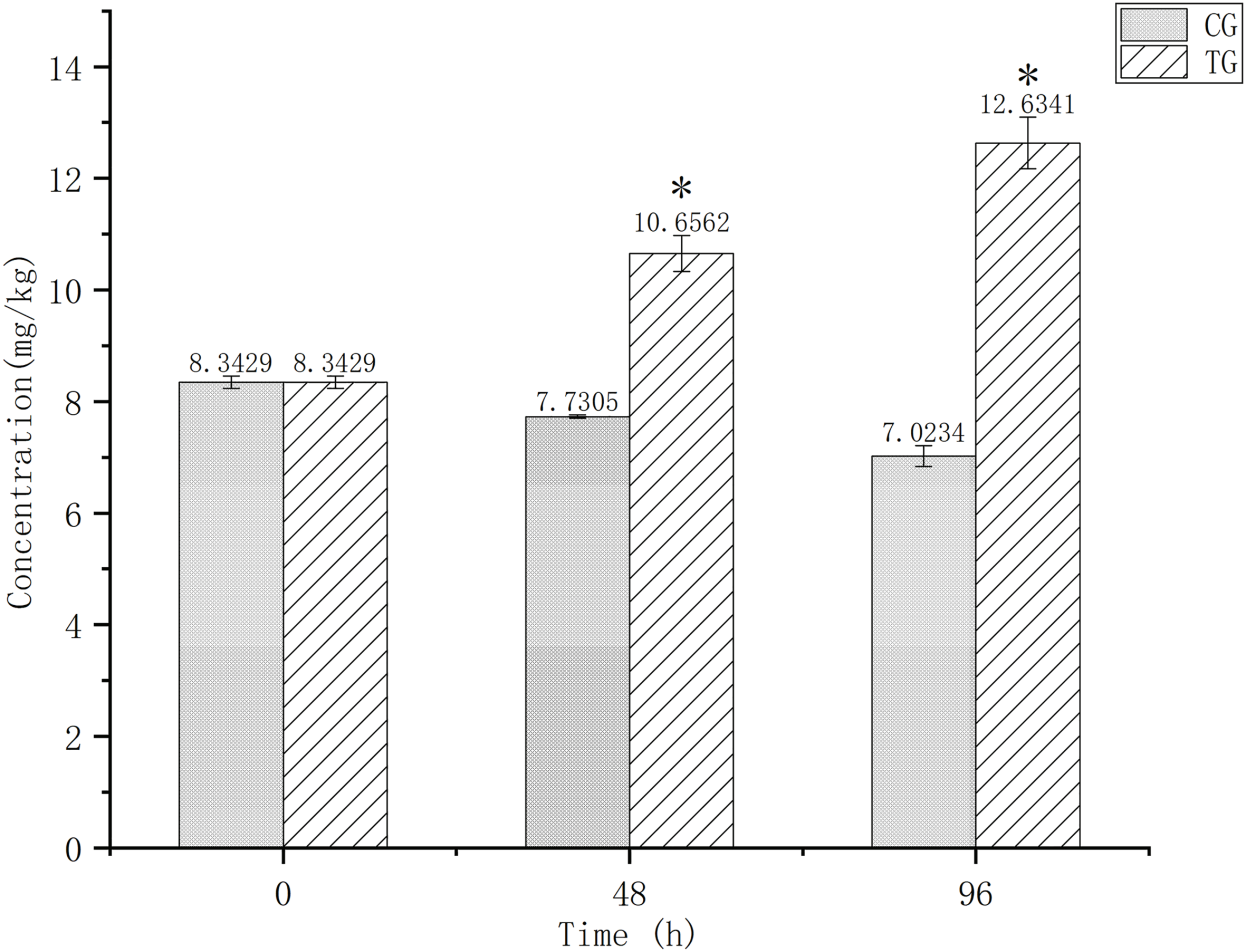
Cadmium (Cd) concentrations in the digestive tissues of *Oratosquilla oratoria*. CG, control group; TG, test group. The significant difference compared to the control group was marked with asterisk (*).

Figure 1 shows Cd contents in the digestive tissues of *O. oratoria* at baseline and after 24 and 96 h of exposure. Baseline concentration was 8.3429 mg/kg and decreased slightly in the control group over time. After 96 h of exposure, Cd concentrations differed significantly between the test (12.6341 mg/kg) and control (7.0234 mg/kg) groups.

### Reliability of Measurements

Quality control samples were used to evaluate the stability of the liquid chromatography-mass spectrometry system. The UHPLC-TOF-MS total ion chromatograms of the quality control samples were compared (Supplementary Figure S1) and showed that the response intensity and retention time of each chromatographic peak generally overlapped, indicating that the variation caused by instrument error was slight throughout the experimental period. Supplementary Figure S2 shows that the relative SD of more than 80% of the peaks detected was below 30%. Quality control samples were clustered closely in the PCA scoring chart (Supplementary Figure S3), confirming the stability and reliability of the mass spectrometry data. One sample in the PCA score chart lay outside Hotelling’s T-squared confidence circle (95%) and was eliminated from subsequent data analysis.

### Metabolic Profile Annotation

After preprocessing, the mass spectrum information was imported into the database for matching, and a total of 589 metabolites were annotated in the Human Metabolome Database. At the subclass level, metabolites were divided into amino acids, glycerophosphoethanolamines, glycerophosphocholines, terpene glycosides, steroidal glycosides, fatty acid esters, carbohydrates, steroid lactones, triterpenoids, fatty acids, glycosphingolipids, and glycerophosphoserines. The amino acids present were mainly arginine, lysine, glycine, and glutamate. The prominent fatty acid esters present were arachidonic acid and carnitine.

### Multivariate Statistical Analysis Results

**Figure 2:**
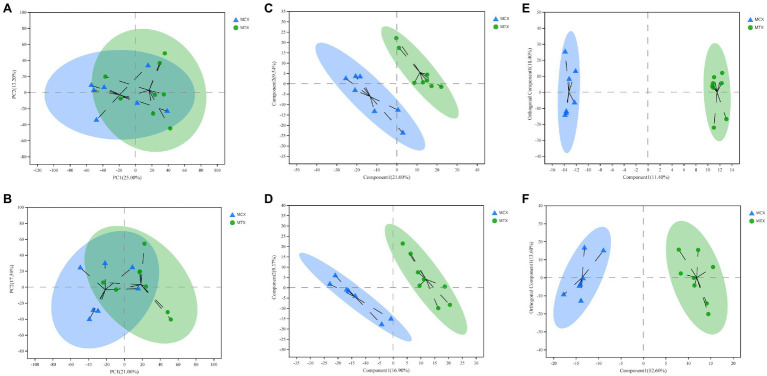
Multivariate statistical analysis of Cd concentrations in the digestive tissues of *O. oratoria*. Principal component analysis (PCA) score chart in positive **(A)** and negative **(B)** modes. Partial least squares discriminant analysis score chart in positive **(C)** and negative **(D)** modes. Orthogonal partial least squares discriminant analysis score chart in positive **(E)** and negative **(F)** modes.

Principal component analysis, OPLS-DA, and PLS-DA were used to compare exposure groups. The PCA score chart showed that all samples were within the 95% Hotelling’s T-squared confidence circle, and the R2 value of all principal components was 0.5100, indicating that the model was statistically significant. Test and control samples tended to separate, but there were overlapping areas. We used OPLS-DA and PLS-DA to achieve a better presentation of differences between groups (Figure 2). Test and control samples separated better under these analyses, and better intra-group aggregation was observed. Table 1 lists model parameters in positive and negative ion modes. R2Y (cumulative) and Q2Y (cumulative) expressed that the model was stable and reliable and had sufficient predictive ability. Response permutation testing was then used to evaluate whether the OPLS-DA and PLS-DA models were overfitting. With the exception of the Q2 value of the OPLS-DA model in POS (0.07), all values were below 0.05, indicating that the model was not overfitting. Overall, the metabolites were clearly separated and the PLS model was better than the OPLS model.

**Table 1:** Model parameters of multivariate statistical analysis on POS and NEG.

	OPLS-DA		PLS-DA	
	R2Y (cum)	Q2Y (cum)	R2Y (cum)	Q2Y (cum)
POS	0.996	0.438	0.952	0.507
NEG	0.991	0.548	0.991	0.581

OPLS-DA, orthogonal partial least squares discriminant analysis; PLS-DA, partial least squares discriminant analysis; POS, positive mode; NEG, negative mode; and cum, cumulative.

### Differential Metabolite Analysis

**Figure 3:**
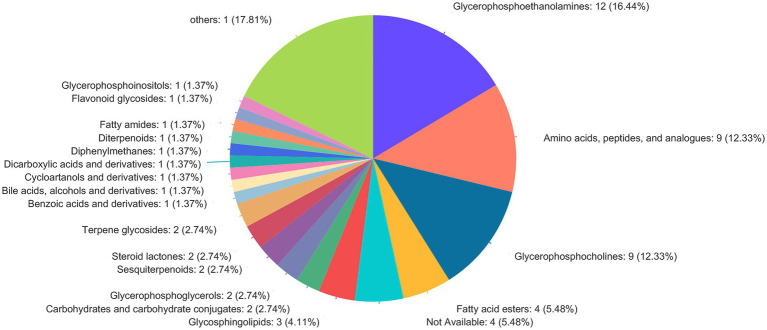
Compound classification in the Human Metabolome Database (HMDB). The different colors in each pie chart in the figure represent different HMDB categories, and the area represents the relative proportion of metabolites in that category.

**Figure 4:**
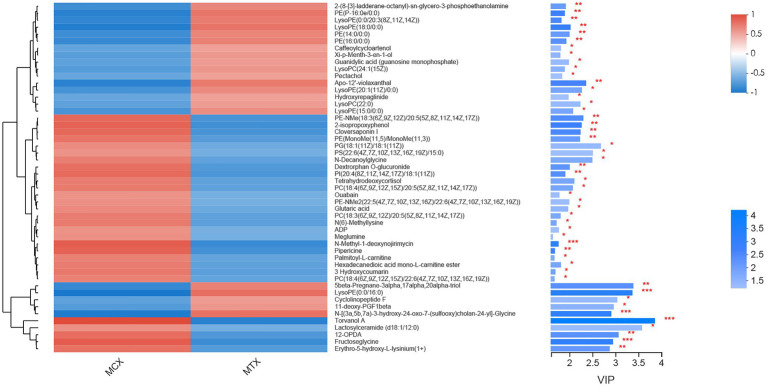
Fifty metabolites with the highest variable importance in projection (VIP) spectrograms under hierarchical cluster analysis. The metabolite cluster dendrogram is on the left. The closer the branch, the closer the expression patterns of all metabolites in different groups; on the right is the VIP bar graph of metabolites. The length of the bar represents the contribution value of the metabolite to the difference between the two groups. The default value is not less than 1. The larger the value, the greater the difference between the two groups. The color of the bar indicates that the metabolites are significantly different (*P*-value) in the two sets of samples. *P*-value < 0.001 was marked as ^***^, *P*-value < 0.01 was marked as ^**^, and *P*-value < 0.05 was marked as ^*^.

The screening of differential metabolites combined single-factor analysis and multivariate statistical analysis using Student’s *t*-test (*p* < 0.05) and variable importance in projection scores > 1. A total of 91 differential metabolites were annotated (Figure 3), mainly including 12 glycerophosphoethanolamines, nine amino acids and peptides, nine glycerophosphocholines, four fatty acid esters, and three glycosphingolipids, etc. Besides, 28 and 63 metabolites were differentially upregulated (fold-change > 1; test vs. control group) and downregulated (fold-change < 1; test vs. control group), respectively. Hierarchical clustering and variable importance in projection scores were used to test the degree of correlation between metabolites and trends in expression changes and showed notable differences between metabolites from *O. oratoria* of the test and control groups (Figure 4).

Metabolite species and the functional pathways involved were annotated using the KEGG database. Metabolites may share KEGG compound identification and each metabolite may be involved in multiple biological pathways. The 22 differential metabolites were annotated as 20 KEGG compounds and are listed in Table 2 with their corresponding pathways (Table 2). About 25 functional pathways were considered related to Cd stress.

**Table 2:** Differential metabolites identified from the KEGG database.

Pathway ID	Description	Metabolites KEGG compound ID
map00071	Fatty acid degradation	C00489; C02990
map00190	Oxidative phosphorylation	C00008
map00230	Purine metabolism	C00008
map00260	Glycine, serine, and threonine metabolism	C06231
map00310	Lysine degradation	C00489
map00564	Glycerophospholipid metabolism	C04230; C00157
map00590	Arachidonic acid metabolism	C00157
map00591	Linoleic acid metabolism	C00157
map00592	Alpha-Linolenic acid metabolism	C01226; C00157
map00600	Sphingolipid metabolism	C01290; C06125; C02686
map01212	Fatty acid metabolism	C02990
map02010	ABC transporters	C01606
map04068	FoxO signaling pathway	C00008
map04080	Neuroactive ligand-receptor interaction	C03958; C00008
map04142	Lysosome	C00008
map04152	AMPK signaling pathway	C00008
map04611	Platelet activation	C00008
map04723	Retrograde endocannabinoid signaling	C00157
map04742	Taste transduction	C00008
map04924	Renin secretion	C00008
map04931	Insulin resistance	C02571
map04976	Bile secretion	C01443
map05012	Parkinson’s disease	C00008
map05231	Choline metabolism in cancer	C04230; C00157
map01100	Metabolic pathways	C01226; C06231; C00008; C09629; C00157; C01290; C06125; C01606; C02686

The functions of the differential metabolites were estimated from enrichment and topological analyses. The key pathways mainly included sphingolipid, fatty acid degradation, choline metabolism in cancer, glycerophospholipid metabolism, fatty acid degradation, forkhead box O (FoxO) signaling, and oxidative phosphorylation.

## Discussion

The aim of this work is to comprehensively characterize the metabolic disorder caused by short-term (96 h) exposure to Cd^2+^ (0.05 mg/L) in the digestive system of *O. oratoria*. The exposure experiment showed that the background concentration of Cd in the digestive system of *O. oratoria* was high, and the excretion is low under natural conditions. After 96 h of exposure, the Cd concentration continued to increase, indicating the high concentration of Cd by mantis shrimp. This work also supports the specific enrichment of Cd by *O. oratoria* as a crustacean (Ren et al., 2019). We screened the metabolites produced in the digestive tissues of *O. oratoria* under Cd stress using untargeted metabolomics. Metabolic pathway analysis indicated that Cd affected lipid metabolism, energy metabolism, oxidative stress, and signaling molecules and interaction.

Eleven amino acids were differentially regulated; lysine (fructoseglycine, N-decanoylglycine), glycine [N (6)-methyllysine, erythro-5-hydroxy-L-lysinium (1+)], neurotensin 11–13, and aspartyllysine were all downregulated, while glutaminylhydroxyproline was slightly upregulated. Glycine and lysine were involved in glutathione metabolism (Nguyen et al., 2020), which regulated the production of reactive oxygen species. Although the expression of antioxidant enzymes was not measured in this study directly, alterations in the expression of these amino acids might indicate that Cd contributes indirectly to oxidative stress (Liu et al., 2009; Bao et al., 2016). Cd inhibits the scavenging of free radicals and can increase the production of reactive oxygen species, causing oxidative stress and lipid peroxidation in organisms (Szuster-Ciesielska et al., 2000; López et al., 2006). Antioxidant enzymes such as superoxide dismutase, catalase, and reduced glutathione are typically measured as indicators of oxidative stress (Hédiji et al., 2010; Sarma et al., 2018; Lu et al., 2020; Mangal et al., 2020). The increase in free amino acids indicates that osmotic regulation and energy metabolism were affected (Viant et al., 2003; Cappello et al., 2017). The different expression changes of glycine also suggest that they are a defense mechanism against Cd-induced oxidative stress. Similar results to this study were also reported in the digestive glands of mussels exposed to microplastics and petroleum pollutants (Fasulo et al., 2012; Cappello et al., 2021). Ji et al. (2016) reported that Cd modulated the expression of glycine, proline, and tyrosine in shrimp and Zhang et al. (2011) reported that the expression of branched chain amino acids in the gill tissues of clams increased following a 96-h exposure to Cd, indicating impairment of osmotic regulation.

Adenosine diphosphate is essential to energy metabolism and was differentially expressed in the present study. Adenosine diphosphate is involved in multiple biological processes, including signal transduction and nucleotide metabolism. Cd is neurotoxic and can inhibit the hydrolysis of adenosine triphosphate (Barcellos et al., 1994) and reduce its levels by altering mitochondrial membrane permeability, thereby inhibiting the respiratory chain and generating reactive oxygen species (Dorta et al., 2003). Wu et al. (2016) also reported similar findings in energy metabolism in mussels exposed to Cd. Therefore, we speculate that the influence of Cd on energy metabolism and oxidative stress is the basis for its osmotic pressure imbalance, neurotoxicity, and other biological effects.

Glycerophospholipids are the main lipid constituents of cell membranes and changes in phospholipid concentrations indicate alterations in cell membrane composition and permeability, which directly affect physiological cell functions. Therefore, changes in phospholipid contents reflect impairments in lipid metabolism (Zong et al., 2018). Around 26 glycerophospholipids were differentially regulated by Cd in our study; these included 12 glycerophosphoethanolamines, 10 glycerophosphocholines, two glycerophosphoglycerols, one glycerophosphoserine, and one glycerophosphoinositol. These metabolites are involved in lipid metabolism, which has been shown to be impaired in animals and plants exposed to Cd (Chen et al., 2013).

Two glycerophosphocholines were downregulated by Cd exposure and were both identified by the KEGG database as C00157. Glycerophosphocholine is the main constituent of the cell membrane, regulating cell metabolism and signal transduction (Maxfield and Tabas, 2005; Fernández-Cisnal et al., 2018). Cell membranes are particularly vulnerable to peroxidative damage from free radicals and reactive oxygen species; increased lipid peroxidation may lead to loss of membrane integrity (Maxfield and Tabas, 2005). Since the damage to cell membranes caused by changes in glycerophosphocholine has also been reported by metabolomics of the digestive system of fish exposed to mercury (Brandão et al., 2015), it is speculated that this may be a mechanical effect of metal exposure toxicity. Lysophosphatidylcholine (LysoPC) is the product of phospholipase A2 (PLA2) hydrolyzing phosphatidylcholine and is upregulated by Cd in rats (Chen et al., 2018). We found that the two metabolites involved in choline metabolism were downregulated following exposure of *O. oratoria* to Cd. We also found that the metabolite C00157 was involved in arachidonic acid metabolism. PLA2, the precursor of lysophosphatidylcholine, has been shown to affect the production of arachidonic acid (Qu et al., 2017; Guan et al., 2020). Gong et al. (2017) reported that the pathogenesis of Cd-induced DNA damage is related to impairment in the metabolism of lipids and arachidonic acid. Cd can cause arachidonic acid to produce a series of pro-inflammatory eicosanoids and potentially toxic reactive oxygen species (Nanda et al., 2007; Al-Asmari et al., 2018). Choline can interfere with intercellular transport and produce betaine (Fernández-Cisnal et al., 2018). Although, betaine was not differentially expressed in our study (variable importance in projection score: 1.109), it regulates osmotic pressure (Viant et al., 2003) and promotes DNA methylation, which in turn affects gene stability (Ueland, 2011). KEGG annotation showed that betaine was related to ABC transporters and the glycine, serine, and threonine metabolism. Ji et al. (2015) used betaine as a biomarker of Cd stress in clams.

KEGG pathway enrichment analysis showed that sphingolipid metabolism and α-linolenic acid metabolism were affected by Cd. Sphingolipid metabolism was mainly affected by the downregulation of lactosylceramide (d18:1/12:0; variable importance in projection score: 3.85). Lactosylceramide is involved in the synthesis of glucosylceramide to promote cell apoptosis (Lee et al., 2011). The metabolism of α-linolenic acid is mainly affected by 12-oxophytodienoic acid and phosphatidylcholine, which is related to the antioxidant capacity of organisms (Hazman et al., 2015). Wang et al. (2008) reported that Cd interferes with intracellular signal transduction by increasing intracellular calcium concentrations to trigger cell death effectors such as ceramide, resulting in irreversible damage to mitochondria and the endoplasmic reticulum. Cd can increase the levels of endogenous ceramides and activate calcium-dependent calpain, causing apoptosis in renal proximal tubule cells (Thévenod and Lee, 2013). Modulating the expression of adenosine diphosphate dysregulates lysosomes, oxidative phosphorylation, and FoxO signaling. Chen et al. (2021) reported that the JNK-FoxO3a-PUMA pathway is involved in Cd-induced oxidative stress and apoptosis. We therefore speculate that Cd affects *O. oratoria* signal transduction and causes autophagy by impairing energy metabolism and inducing oxidative stress.

## Conclusion

In this work, we characterized the changes of metabolites in the digestive system of *O. oratoria* under the stress of 0.05 mg/L Cd^2+^ for 96 h and the functional pathways that affected them. The results showed that 91 differential metabolites were separated by PLS-DA analysis (VIP > 1) and Student’s *t*-test (*p* < 0.05). The amino acids involved in various metabolic pathways, such as glycerophospholipids and glycerophosphocholines, have undergone significant changes. Among them, LysoPC (22:0) expression was significantly upregulated (FC = 1.36), and 12-OPDA, Lactosylceramide (d18:1/12:0), ADP, and Glutaric acid expression were significantly downregulated (FC < 0.85) in the digestive system of *O. oratoria* under Cd exposure, could be used as potential biomarkers. Metabolic pathway analysis showed that the digestive system of *O. oratoria* produced a defense mechanism against Cd-induced oxidative stress, and osmotic pressure regulation, energy metabolism, signal transduction, lipid metabolism, and choline metabolism were disturbed by Cd. In addition, we speculate that lipid metabolism, oxidative stress, and energy metabolism are the critical pathways that are interfered with by Cd and cause other physiological disorders. Overall, this work initially discussed the toxicological response of *O. oratoria* to Cd stress at the metabolic level, and provided new insights into the potential mechanism. It is necessary to conduct further research to verify the separated differential metabolites and clarify the toxicity mechanism of cadmium to *O. oratoria*.

## Data Availability Statement

The datasets presented in this study can be found in online repositories. The names of the repository/repositories and accession number(s) can be found at: https://www.ebi.ac.uk/metabolights/, MTBLS2817.

## Author Contributions

YX and HL designed research studies, conducted experiments, analyzed data, and drafted the manuscript. DH, LR, and XG provided intellectual input into planning of experiments. FJ, YC, and XL developed the analytical method. CR and JX analyzed the exposure experiment data. XT conducted sample collection and storage. All authors contributed to the article and approved the submitted version.

## Conflict of Interest

The authors declare that the research was conducted in the absence of any commercial or financial relationships that could be construed as a potential conflict of interest.

## Publisher’s Note

All claims expressed in this article are solely those of the authors and do not necessarily represent those of their affiliated organizations, or those of the publisher, the editors and the reviewers. Any product that may be evaluated in this article, or claim that may be made by its manufacturer, is not guaranteed or endorsed by the publisher.

## Footnotes

^1^ http://www.hmdb.ca/

^2^ https://metlin.scripps.edu/

^3^ https://cloud.majorbio.com

^4^ http://www.genome.jp/kegg/

^5^ https://docs.scipy.org/doc/scipy/

## References

[ref1] Al-Asmari A. K. Khan H. A. Manthiri R. A. Al-Khlaiwi A. A. Al-Asmari B. A. Ibrahim K. E. (2018). Protective effects of a natural herbal compound quercetin against snake venom-induced hepatic and renal toxicities in rats. Food Chem. Toxicol. 118, 105–110. 10.1016/j.fct.2018.05.016, PMID: 29751071

[ref2] Bao Y. Liu X. Zhang W. Cao J. Li W. Li C.. (2016). Identification of a regulation network in response to cadmium toxicity using blood clam Tegillarca granosa as model. Sci. Rep. 6:35704. 10.1038/srep35704, PMID: 27760991 PMC5071765

[ref3] Barcellos C. K. Schetinger M. R. Battastini A. M. Silva L. B. Dias R. D. Sarkis J. J. (1994). Inhibitory effect of cadmium acetate on synaptosomal ATP diphosphohydrolase (EC 3.6.1.5; apyrase) from adult rat cerebral cortex. Braz. J. Med. Biol. Res. 27, 1111–1115. PMID: 8000330

[ref4] Bernard A. (2004). Renal dysfunction induced by cadmium: biomarkers of critical effects. Biometals 17, 519–523. 10.1023/B:BIOM.0000045731.75602.b9, PMID: 15688856

[ref5] Boroujerdi A. F. B. Vizcaino M. I. Meyers A. Pollock E. C. Bearden D. W. (2009). NMR-based microbial metabolomics and the temperature-dependent coral pathogen *Vibrio coralliilyticus*. Environ. Sci. Technol. 43, 7658–7664. 10.1021/es901675w, PMID: 19921875

[ref6] Brandão F. Cappello T. Raimundo J. Santos M. A. Maisano M. Mauceri A.. (2015). Unravelling the mechanisms of mercury hepatotoxicity in wild fish (*Liza aurata*) through a triad approach: bioaccumulation, metabolomic profiles and oxidative stress. Metallomics 7, 1352–1363. 10.1039/C5MT00090D, PMID: 26084244

[ref7] Cappello T. (2020). “NMR-based metabolomics of aquatic organisms,” in eMagRes. *Vol*. 8. eds. Harris R. K. Wasylishen R. L. 81–100.

[ref8] Cappello T. De Marco G. Conti G. O. Giannetto A. Ferrante M. Mauceri A.. (2021). Time-dependent metabolic disorders induced by short-term exposure to polystyrene microplastics in the Mediterranean mussel *Mytilus galloprovincialis*. Ecotoxicol. Environ. Saf. 209:111780. 10.1016/j.ecoenv.2020.111780, PMID: 33352432

[ref9] Cappello T. Maisano M. Mauceri A. Fasulo S. (2017). 1H NMR-based metabolomics investigation on the effects of petrochemical contamination in posterior adductor muscles of caged mussel *Mytilus galloprovincialis*. Ecotoxicol. Environ. Saf. 142, 417–422. 10.1016/j.ecoenv.2017.04.040, PMID: 28454054

[ref10] Chaleckis R. Meister I. Zhang P. Wheelock C. E. (2019). Challenges, progress and promises of metabolite annotation for LC-MS-based metabolomics. Curr. Opin. Biotechnol. 55, 44–50. 10.1016/j.copbio.2018.07.010, PMID: 30138778

[ref11] Chen J. Chen D. Li J. Liu Y. Gu X. Teng X. (2021). Cadmium-induced oxidative stress and immunosuppression mediated mitochondrial apoptosis via JNK-FoxO3a-PUMA pathway in common carp (*Cyprinus carpio L*.) gills. Aquat. Toxicol. 233:105775. 10.1016/j.aquatox.2021.105775, PMID: 33631492

[ref12] Chen Q. L. Gong Y. Luo Z. Zheng J. L. Zhu Q. L. (2013). Differential effect of waterborne cadmium exposure on lipid metabolism in liver and muscle of yellow catfish *Pelteobagrus fulvidraco*. Aquat. Toxicol. 142–143, 380–386. 10.1016/j.aquatox.2013.09.011, PMID: 24095957

[ref13] Chen S. Zhang M. Bo L. Li S. Hu L. Zhao X.. (2018). Metabolomic analysis of the toxic effect of chronic exposure of cadmium on rat urine. Environ. Sci. Pollut. Res. Int. 25, 3765–3774. 10.1007/s11356-017-0774-8, PMID: 29168138

[ref14] Dorta D. J. Leite S. DeMarco K. C. Prado I. M. R. Rodrigues T. Mingatto F. E.. (2003). A proposed sequence of events for cadmium-induced mitochondrial impairment. J. Inorg. Biochem. 97, 251–257. 10.1016/S0162-0134(03)00314-3, PMID: 14511887

[ref15] Đukić-Ćosić D. Baralić K. Javorac D. Djordjevic A. B. Bulat Z. (2020). An overview of molecular mechanisms in cadmium toxicity. Curr. Opin. Toxicol. 19, 56–62. 10.1016/j.cotox.2019.12.002

[ref16] Fasulo S. Iacono F. Cappello T. Corsaro C. Maisano M. D’Agata A.. (2012). Metabolomic investigation of *Mytilus galloprovincialis* (Lamarck 1819) caged in aquatic environments. Ecotoxicol. Environ. Saf. 84, 139–146. 10.1016/j.ecoenv.2012.07.001, PMID: 22818846

[ref17] Fernández-Cisnal R. García-Sevillano M. A. García-Barrera T. Gómez-Ariza J. L. Abril N. (2018). Metabolomic alterations and oxidative stress are associated with environmental pollution in *Procambarus clarkii*. Aquat. Toxicol. 205, 76–88. 10.1016/j.aquatox.2018.10.005, PMID: 30343212

[ref18] Fu B. Zhao J. Peng W. Wu H. Zhang Y. (2017). Resveratrol rescues cadmium-induced mitochondrial injury by enhancing transcriptional regulation of PGC-1α and SOD2 via the Sirt3/FoxO3a pathway in TCMK-1 cells. Biochem. Biophys. Res. Commun. 486, 198–204. 10.1016/j.bbrc.2017.03.027, PMID: 28286268

[ref19] Gao X. Zhou F. Chen C.-T. A. (2014). Pollution status of the Bohai Sea: an overview of the environmental quality assessment related trace metals. Environ. Int. 62, 12–30. 10.1016/j.envint.2013.09.019, PMID: 24161379

[ref20] Gill S. S. Tuteja N. (2010). Reactive oxygen species and antioxidant machinery in abiotic stress tolerance in crop plants. Plant Physiol. Biochem. 48, 909–930. 10.1016/j.plaphy.2010.08.016, PMID: 20870416

[ref21] Godt J. Scheidig F. Grosse-Siestrup C. Esche V. Brandenburg P. Reich A.. (2006). The toxicity of cadmium and resulting hazards for human health. J. Occup. Med. Toxicol. 1:22. 10.1186/1745-6673-1-22, PMID: 16961932 PMC1578573

[ref22] Gong P. Chang X. Chen X. Bai X. Wen H. Pi S.. (2017). Metabolomics study of cadmium-induced diabetic nephropathy and protective effect of caffeic acid phenethyl ester using UPLC–Q-TOF-MS combined with pattern recognition. Environ. Toxicol. Pharmacol. 54, 80–92. 10.1016/j.etap.2017.06.021, PMID: 28704754

[ref23] Guan T. Xin Y. Zheng K. Wang R. Zhang X. Jia S.. (2020). Metabolomics analysis of the effects of quercetin on renal toxicity induced by cadmium exposure in rats. BioMetals 34, 33–48. 10.1007/s10534-020-00260-2, PMID: 33033991

[ref24] Hazman M. Hause B. Eiche E. Nick P. Riemann M. (2015). Increased tolerance to salt stress in OPDA-deficient rice allene oxide cyclase mutants is linked to an increased ROS-scavenging activity. J. Exp. Bot. 66, 3339–3352. 10.1093/jxb/erv142, PMID: 25873666 PMC4449546

[ref25] Hédiji H. Djebali W. Cabasson C. Maucourt M. Baldet P. Bertrand A.. (2010). Effects of long-term cadmium exposure on growth and metabolomic profile of tomato plants. Ecotoxicol. Environ. Saf. 73, 1965–1974. 10.1016/j.ecoenv.2010.08.014, PMID: 20846723

[ref26] Henson M. C. Chedrese P. J. (2004). Endocrine disruption by cadmium, a common environmental toxicant with paradoxical effects on reproduction. Exp. Biol. Med. 229, 383–392. 10.1177/153537020422900506, PMID: 15096650

[ref27] Holmstrand H. Gadomski D. Mandalakis M. Tysklind M. Irvine R. Andersson P.. (2006). Origin of PCDDs in ball clay assessed with compound-specific chlorine isotope analysis and radiocarbon dating. Environ. Sci. Technol. 40, 3730–3735. 10.1021/es0602142, PMID: 16830534

[ref28] Hu S. Su Z. Jiang J. Huang W. Liang X. Hu J.. (2016). Lead, cadmium pollution of seafood and human health risk assessment in the coastline of the southern China. Stoch. Env. Res. Risk A. 30, 1379–1386. 10.1007/s00477-015-1139-9

[ref29] Ji C. Lu Z. Xu L. Li F. Cong M. Shan X.. (2019). Evaluation of mitochondrial toxicity of cadmium in clam *Ruditapes philippinarum* using iTRAQ-based proteomics. Environ. Pollut. 251, 802–810. 10.1016/j.envpol.2019.05.046, PMID: 31125810

[ref30] Ji C. Wu H. Zhou M. Zhao J. (2015). Multiple biomarkers of biological effects induced by cadmium in clam *Ruditapes philippinarum*. Fish Shellfish Immunol. 44, 430–435. 10.1016/j.fsi.2015.03.024, PMID: 25804494

[ref31] Ji C. Yu D. Wang Q. Li F. Zhao J. Wu H. (2016). Impact of metal pollution on shrimp *Crangon affinis* by NMR-based metabolomics. Mar. Pollut. Bull. 106, 372–376. 10.1016/j.marpolbul.2016.02.052, PMID: 26920426

[ref32] Kim S. Yoon D. Lee M. Yoon C. Kim S. (2016). Metabolic responses in zebrafish (*Danio rerio*) exposed to zinc and cadmium by nuclear magnetic resonance -based metabolomics. Chem. Ecol. 32, 136–148. 10.1080/02757540.2015.1125891

[ref33] Kumar S. Sharma A. (2019). Cadmium toxicity: effects on human reproduction and fertility. Rev. Environ. Health 34, 327–338. 10.1515/reveh-2019-0016, PMID: 31129655

[ref34] Lee W. K. Torchalski B. Kohistani N. Thévenod F. (2011). ABCB1 protects kidney proximal tubule cells against cadmium-induced apoptosis: roles of cadmium and ceramide transport. Toxicol. Sci. 121, 343–356. 10.1093/toxsci/kfr071, PMID: 21436125

[ref35] Li M. Zhang C. Xu B. Xue Y. Ren Y. (2020). A comparison of GAM and GWR in modelling spatial distribution of Japanese mantis shrimp (*Oratosquilla oratoria*) in coastal waters. Estuar. Coast. Shelf Sci. 244:106928. 10.1016/j.ecss.2020.106928

[ref36] Liu Y. Cui Z. (2010). The complete mitochondrial genome of the mantid shrimp *Oratosquilla oratoria* (Crustacea: Malacostraca: *Stomatopoda*): novel non-coding regions features and phylogenetic implications of the *Stomatopoda*. Comp. Biochem. Physiol. Part D Genomics Proteomics 5, 190–198. 10.1016/j.cbd.2010.04.001, PMID: 20510661

[ref37] Liu H. Li H. Zhang X. Gong X. Xu Y. (2021). Metabolomics comparison of metabolites and functional pathways in the gills of *Chlamys farreri* under cadmium exposure. Environ. Toxicol. Pharmacol. 86:103683. 10.1016/j.etap.2021.103683, PMID: 34052434

[ref38] Liu J. Qu W. Kadiiska M. B. (2009). Role of oxidative stress in cadmium toxicity and carcinogenesis. Toxicol. Appl. Pharmacol. 238, 209–214. 10.1016/j.taap.2009.01.029, PMID: 19236887 PMC4287357

[ref39] López E. Arce C. Oset-Gasque M. J. Cañadas S. González M. P. (2006). Cadmium induces reactive oxygen species generation and lipid peroxidation in cortical neurons in culture. Free Radic. Biol. Med. 40, 940–951. 10.1016/j.freeradbiomed.2005.10.062, PMID: 16540389

[ref40] Lou F. Gao T. Cai S. Han Z. (2018). De novo assembly and annotation of the whole transcriptome of *Oratosquilla oratoria*. Mar. Genomics 38, 17–20. 10.1016/j.margen.2017.08.003, PMID: 28870633

[ref41] Lu Z. Wang S. Ji C. Shan X. Wu H. (2020). Evaluation of metal pollution-induced biological effects in Chinese shrimp *Fenneropenaeus chinensis* by NMR-based metabolomics. Mar. Pollut. Bull. 150:110688. 10.1016/j.marpolbul.2019.110688, PMID: 31677417

[ref42] Mangal V. Nguyen T. Q. Fiering Q. Guéguen C. (2020). An untargeted metabolomic approach for the putative characterization of metabolites from *Scenedesmus obliquus* in response to cadmium stress. Environ. Pollut. 266:115123. 10.1016/j.envpol.2020.115123, PMID: 32688078

[ref43] Maxfield F. R. Tabas I. (2005). Role of cholesterol and lipid organization in disease. Nature 438, 612–621. 10.1038/nature04399, PMID: 16319881

[ref44] Messaoudi I. Barhoumi S. Saïd K. Kerken A. (2009). Study on the sensitivity to cadmium of marine fish *Salaria basilisca* (Pisces: Blennidae). J. Environ. Sci. 21, 1620–1624. 10.1016/S1001-0742(08)62464-X, PMID: 20108699

[ref45] Milreu P. V. Klein C. C. Cottret L. Acuña V. Birmelé E. Borassi M.. (2013). Telling metabolic stories to explore metabolomics data: a case study on the yeast response to cadmium exposure. Bioinformatics 30, 61–70. 10.1093/bioinformatics/btt597, PMID: 24167155 PMC3866556

[ref46] Mwamba T. M. Islam F. Ali B. Lwalaba J. L. W. Gill R. A. Zhang F.. (2020). Comparative metabolomic responses of low- and high-cadmium accumulating genotypes reveal the cadmium adaptive mechanism in *Brassica napus*. Chemosphere 250:126308. 10.1016/j.chemosphere.2020.126308, PMID: 32135439

[ref47] Nanda B. L. Nataraju A. Rajesh R. Rangappa K. S. Shekar M. A. Vishwanath B. S. (2007). PLA2 mediated arachidonate free radicals: PLA2 inhibition and neutralization of free radicals by anti-oxidants—a new role as anti-inflammatory molecule. Curr. Top. Med. Chem. 7, 765–777. 10.2174/156802607780487623, PMID: 17456040

[ref48] Nguyen T. V. Ragg N. L. C. Alfaro A. C. Zamora L. N. (2020). Physiological stress associated with mechanical harvesting and transport of cultured mussels (*Perna canaliculus*): a metabolomics approach. Aquaculture 529:735657. 10.1016/j.aquaculture.2020.735657

[ref49] Nicholson J. K. Lindon J. C. Holmes E. (1999). ‘Metabonomics’: understanding the metabolic responses of living systems to pathophysiological stimuli via multivariate statistical analysis of biological NMR spectroscopic data. Xenobiotica 11, 1181–1189. 10.1080/004982599238047, PMID: 10598751

[ref50] Olsvik P. A. Aulin M. Samuelsen O. B. Hannisdal R. Agnalt A. Lunestad B. T. (2019). Whole-animal accumulation, oxidative stress, transcriptomic and metabolomic responses in the pink shrimp (*Pandalus montagui*) exposed to teflubenzuron. J. Appl. Toxicol. 39, 485–497. 10.1002/jat.3739, PMID: 30345541

[ref51] Qu Y. Zhang H. L. Zhang X. P. Jiang H. L. (2017). Arachidonic acid attenuates brain damage in a rat model of ischemia/reperfusion by inhibiting inflammatory response and oxidative stress. Hum. Exp. Toxicol. 37, 135–141. 10.1177/0960327117692134, PMID: 29233001

[ref52] Ren X. Wang X. Liu P. Li J. (2019). Bioaccumulation and physiological responses in juvenile *Marsupenaeus japonicus* exposed to cadmium. Aquat. Toxicol. 214:105255. 10.1016/j.aquatox.2019.105255, PMID: 31325645

[ref53] Sarma S. N. Saleem A. Lee J. Y. Tokumoto M. Hwang G. W. Man Chan H.. (2018). Effects of long-term cadmium exposure on urinary metabolite profiles in mice. J. Toxicol. Sci. 43, 89–100. 10.2131/jts.43.89, PMID: 29479038

[ref54] Song Y. Chai T. Yin Z. Zhang X. Zhang W. Qian Y.. (2018). Stereoselective effects of ibuprofen in adult zebrafish (*Danio rerio*) using UPLC-TOF/MS-based metabolomics. Environ. Pollut. 241, 730–739. 10.1016/j.envpol.2018.06.009, PMID: 29908497

[ref55] Sugiura Y. Kashiba M. Maruyama K. Hoshikawa K. Sasaki R. Saito K.. (2005). Cadmium exposure alters metabolomics of sulfur-containing amino acids in rat testes. Antioxid. Redox Signal. 7, 781–787. 10.1089/ars.2005.7.781, PMID: 15890025

[ref56] Sumner L. W. Mendes P. Dixon R. A. (2003). Plant metabolomics: large-scale phytochemistry in the functional genomics era. Phytochemistry 62, 817–836. 10.1016/S0031-9422(02)00708-2, PMID: 12590110

[ref57] Sun L. Cao X. Tan C. Deng Y. Cai R. Peng X.. (2020). Analysis of the effect of cadmium stress on root exudates of *Sedum plumbizincicola* based on metabolomics. Ecotoxicol. Environ. Saf. 205:111152. 10.1016/j.ecoenv.2020.111152, PMID: 32846297

[ref58] Szuster-Ciesielska A. Stachura A. Słotwińska M. Kamińska T. Śnieżko R. Paduch R.. (2000). The inhibitory effect of zinc on cadmium-induced cell apoptosis and reactive oxygen species (ROS) production in cell cultures. Toxicology 145, 159–171. 10.1016/S0300-483X(00)00144-X, PMID: 10771141

[ref59] Thévenod F. Lee W. K. (2013). Cadmium and cellular signaling cascades: interactions between cell death and survival pathways. Arch. Toxicol. 87, 1743–1786. 10.1007/s00204-013-1110-9, PMID: 23982889

[ref60] Ueland P. M. (2011). Choline and betaine in health and disease. J. Inherit. Metab. Dis. 34, 3–15. 10.1007/s10545-010-9088-4, PMID: 20446114

[ref61] Venter L. Loots D. T. Mienie L. J. Rensburg P. J. J. V. Mason S. Vosloo A.. (2018). Uncovering the metabolic response of abalone (*Haliotis midae*) to environmental hypoxia through metabolomics. Metabolomics 14:49. 10.1007/s11306-018-1346-8, PMID: 30830330

[ref62] Viant M. R. Rosenblum E. S. Tieerdema R. S. (2003). NMR-based metabolomics: a powerful approach for characterizing the effects of environmental stressors on organism health. Environ. Sci. Technol. 37, 4982–4989. 10.1021/es034281x, PMID: 14620827

[ref63] Vinaixa M. Schymanski E. L. Neumann S. Navarro M. Salek R. M. Yanes O. (2016). Mass spectral databases for LC/MS- and GC/MS-based metabolomics: state of the field and future prospects. TRAC Trend. Anal. Chem. 78, 23–35. 10.1016/j.trac.2015.09.005

[ref64] Wang S. H. Shih Y. L. Ko W. C. Wei Y. H. Shih C. M. (2008). Cadmium-induced autophagy and apoptosis are mediated by a calcium signaling pathway. Cell. Mol. Life Sci. 65, 3640–3652. 10.1007/s00018-008-8383-9, PMID: 18850067 PMC11131605

[ref65] Wang X. Sun G. Feng T. Zhang J. Geng M. (2019). Sodium oligomannate therapeutically remodels gut microbiota and suppresses gut bacterial amino acids-shaped neuroinflammation to inhibit Alzheimer’s disease progression. Cell Res. 29, 1–17. 10.1038/s41422-019-0216-x, PMID: 31488882 PMC6796854

[ref66] Wu Y. Chen H. Wu Z. Pan Y. Chen P. (2018). Concentrations and risk evaluation of four kinds of heavy metals in fishing aquatic products of the central and northern sea areas of Fujian Province. J. Fish. Res. 40, 478–489. 10.14012/j.cnki.fjsc.2018.06.008

[ref67] Wu H. Xu L. Ji C. Yu D. (2016). Proteomic and metabolomic responses in D-shape larval mussels *Mytilus galloprovincialis* exposed to cadmium and arsenic. Fish Shellfish Immunol. 58, 514–520. 10.1016/j.fsi.2016.09.064, PMID: 27702675

[ref68] Xu X. Yao H. Meng X. Gan H. Liu T. Fang T.. (2019). Heavy metal pollution and ecological risk assessment of sea water, surface sediments and aquatic products in the sea area near Lianyungang. Trans. Oceanol. Limnol. 170, 112–118. 10.13984/j.cnki.cn37-1141.2019.05.014

[ref69] Yan H. Cui X. Shen X. Wang L. Jiang L. Liu H.. (2018). De novo transcriptome analysis and differentially expressed genes in the ovary and testis of the Japanese mantis shrimp *Oratosquilla oratoria* by RNA-Seq. Comp. Biochem. Physiol. Part D Genomics Proteomics 26, 69–78. 10.1016/j.cbd.2018.04.001, PMID: 29702368

[ref70] Zhang D. Ding G. Ge B. Zhang H. Tang B. Yang G. (2014). Comparative phylogeography of two marine species of crustacean: recent divergence and expansion due to environmental changes. Gene 550, 141–147. 10.1016/j.gene.2014.08.006, PMID: 25106858

[ref71] Zhang L. Liu X. You L. Zhou D. Yu J. Zhao J.. (2011). Toxicological effects induced by cadmium in gills of manila clam *Ruditapes philippinarum* using NMR-based metabolomics. Clean Soil Air Water 39, 989–995. 10.1002/clen.201100208

[ref72] Zhang N. Mu W. Zhang S. Fang H.-N. Zhu L. Zheng C. (2016). Test and evaluation of heavy metals in seafood from Zhoushan. Guangzhou Chemical Industry (CN) 44, 101–103.

[ref73] Zhang H. Zhai Y. (2019). Integrated transcriptomic and proteomic analyses of the tissues from the digestive gland of *Chlamys farreri* following cadmium exposure. J. Cell. Biochem. 121, 974–983. 10.1002/jcb.29254, PMID: 31696969

[ref74] Zhao Y. Kang X. Ning J. Yuxiu Z. Sheng X. (2019). Species analysis and distribution characteristic of cadmium and arsenic in the different edible tissues of *Oratosquilla oratoria*. Chin. J. Food Sci. 41, 282–287. 10.7506/spkx1002-6630-20190119-229

[ref75] Zong L. Xing J. Liu S. Liu Z. Song F. (2018). Cell metabolomics reveals the neurotoxicity mechanism of cadmium in PC12 cells. Ecotoxicol. Environ. Saf. 147, 26–33. 10.1016/j.ecoenv.2017.08.028, PMID: 28822947

