# Supplementary figures and images for: Metabolomic Alterations in the Digestive System of the Mantis Shrimp Oratosquilla oratoria Following Short-Term Exposure to Cadmium
Source: Front Physiol. 2021 Aug 5;12:706579. doi: 10.3389/fphys.2021.706579 (PMC8374601; doi:10.3389/fphys.2021.706579)

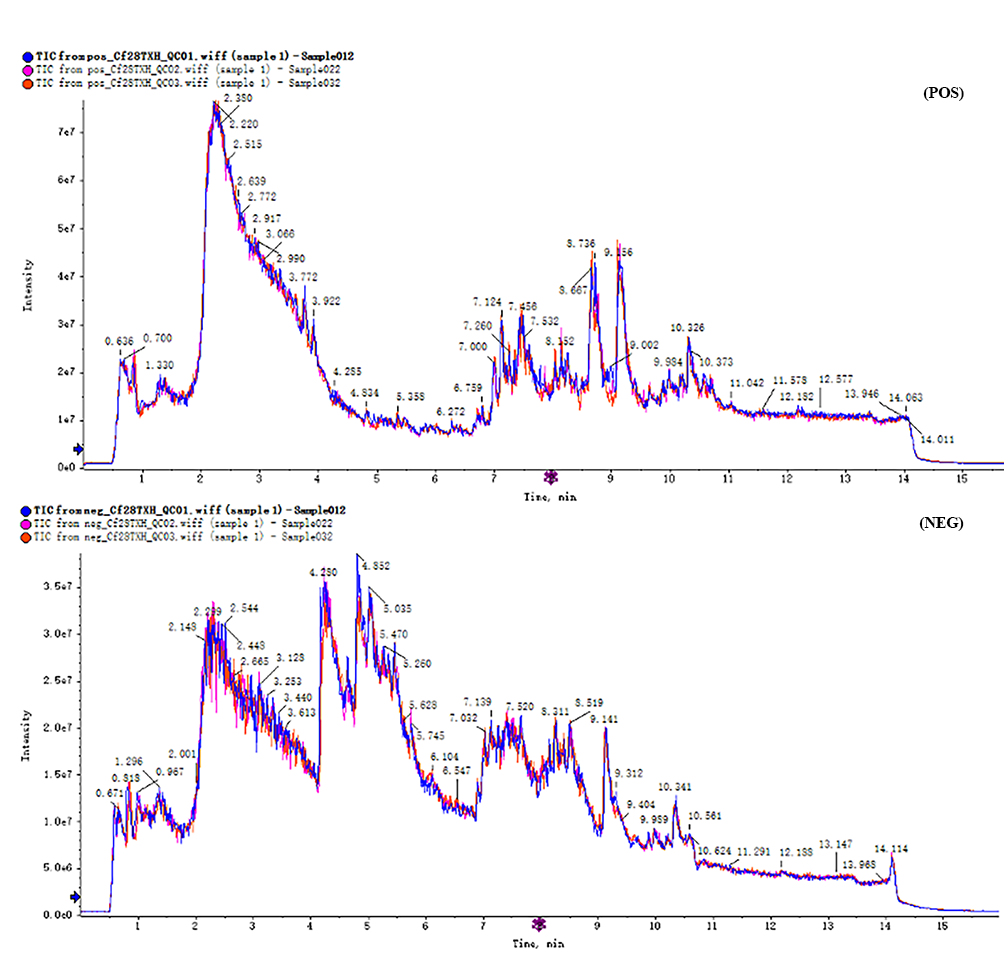

Supplement: Supplementary file 1 [file Image_1.tif]

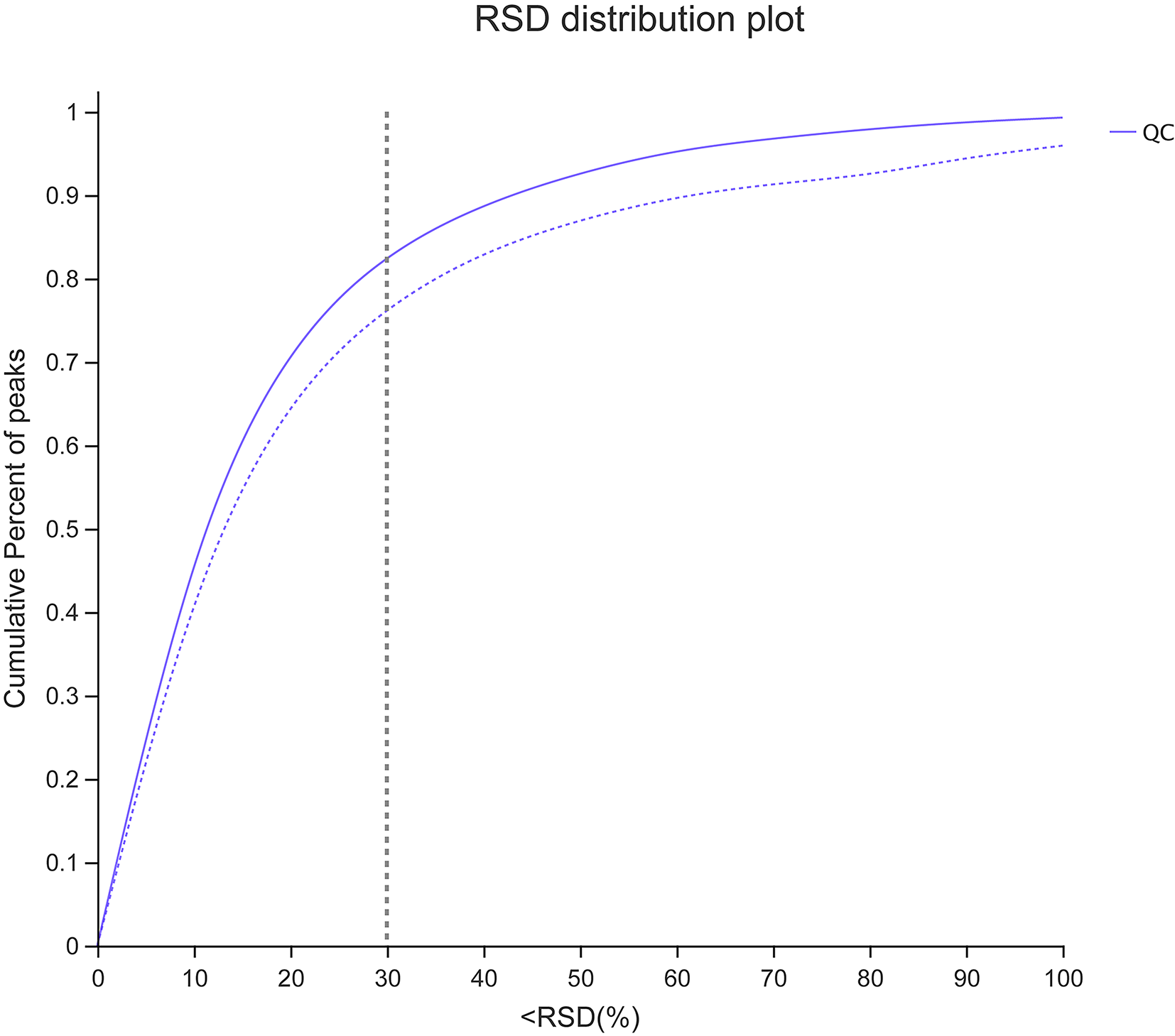

Supplement: Supplementary file 2 [file Image_2.tif]

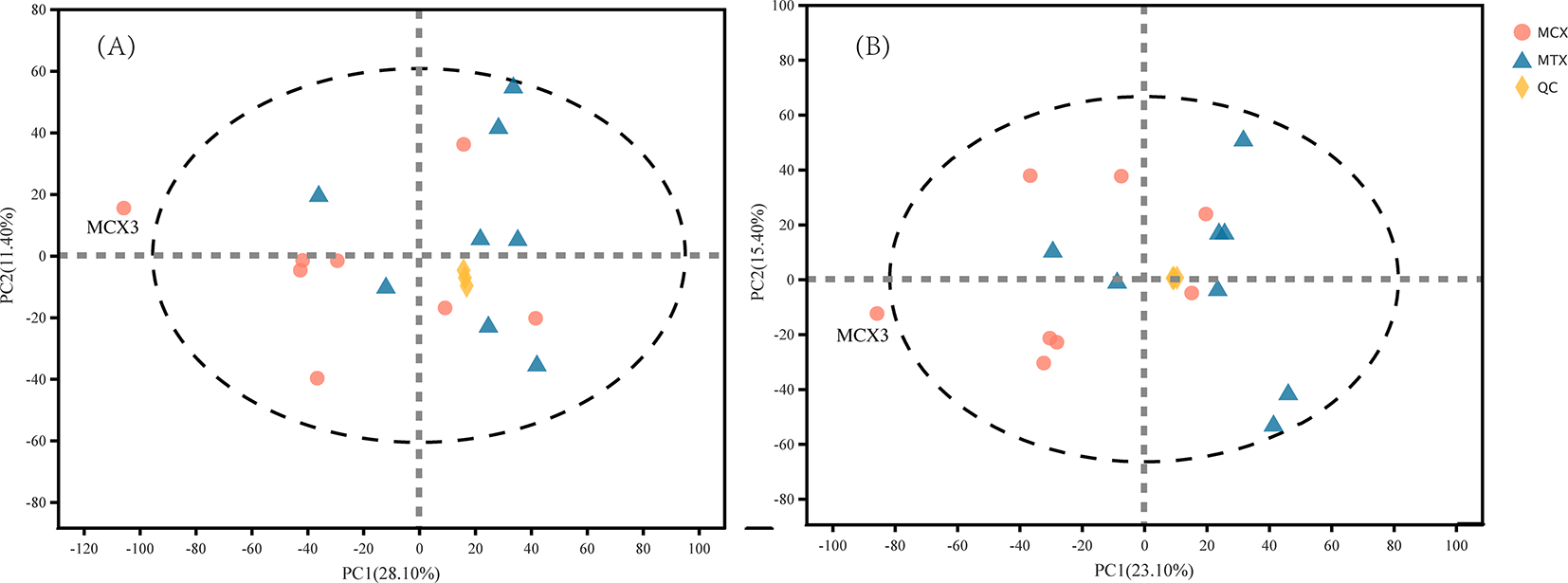

Supplement: Supplementary file 3 [file Image_3.tif]
